# Broad Spectrum Antiviral Activity of Favipiravir (T-705): Protection from Highly Lethal Inhalational Rift Valley Fever

**DOI:** 10.1371/journal.pntd.0002790

**Published:** 2014-04-10

**Authors:** Amy L. Caroline, Diana S. Powell, Laura M. Bethel, Tim D. Oury, Douglas S. Reed, Amy L. Hartman

**Affiliations:** 1 Regional Biocontainment Laboratory, Center for Vaccine Research, University of Pittsburgh, Pittsburgh, Pennsylvania, United States of America; 2 Department of Pathology, University of Pittsburgh School of Medicine, Pittsburgh, Pennsylvania, United States of America; 3 Department of Immunology, University of Pittsburgh School of Medicine, Pittsburgh, Pennsylvania, United States of America; 4 Department of Infectious Diseases and Microbiology, University of Pittsburgh Graduate School of Public Health, Pittsburgh, Pennsylvania, United States of America; U.S. Naval Medical Research Unit No. 2, Indonesia

## Abstract

**Background:**

Development of antiviral drugs that have broad-spectrum activity against a number of viral infections would be of significant benefit. Due to the evolution of resistance to currently licensed antiviral drugs, development of novel anti-influenza drugs is in progress, including Favipiravir (T-705), which is currently in human clinical trials. T-705 displays broad-spectrum *in vitro* activity against a number of viruses, including Rift Valley Fever virus (RVFV). RVF is an important neglected tropical disease that causes human, agricultural, and economic losses in endemic regions. RVF has the capacity to emerge in new locations and also presents a potential bioterrorism threat. In the current study, the *in vivo* efficacy of T-705 was evaluated in Wistar-Furth rats infected with the virulent ZH501 strain of RVFV by the aerosol route.

**Methodology/Principal Findings:**

Wistar-Furth rats are highly susceptible to a rapidly lethal disease after parenteral or inhalational exposure to the pathogenic ZH501 strain of RVFV. In the current study, two experiments were performed: a dose-determination study and a delayed-treatment study. In both experiments, all untreated control rats succumbed to disease. Out of 72 total rats infected with RVFV and treated with T-705, only 6 succumbed to disease. The remaining 66 rats (92%) survived lethal infection with no significant weight loss or fever. The 6 treated rats that succumbed survived significantly longer before succumbing to encephalitic disease.

**Conclusions/Significance:**

Currently, there are no licensed antiviral drugs for treating RVF. Here, T-705 showed remarkable efficacy in a highly lethal rat model of Rift Valley Fever, even when given up to 48 hours post-infection. This is the first study to show protection of rats infected with the pathogenic ZH501 strain of RVFV. Our data suggest that T-705 has potential to be a broad-spectrum antiviral drug.

## Author Summary

Broad-spectrum antiviral drugs are preferred because they have the capacity to treat a range of viral illnesses rather than just one. Food and Drug Administration (FDA) approval of antiviral drugs to treat neglected tropical diseases is difficult to obtain due to ethical and logistical considerations when conducting human clinical trials. Rift Valley Fever (RVF) is an endemic tropical disease that causes human morbidity and mortality, as well as economic damage to the livestock industry. There are no licensed antiviral drugs to treat RVF. In this study, we found that a novel anti-influenza drug, Favipiravir (T-705), is able to prevent lethal RVF in rats, and therefore shows promise as a broad-spectrum antiviral treatment.

## Introduction

Food and Drug Administration (FDA)-licensed antiviral drugs generally target one virus or a small group of closely-related viruses. Development of a broad-spectrum antiviral drug that could treat a range of different viral infections would be useful, especially if the drug acts against emerging viruses or those that could be used as a bioweapon. Successful antiviral drugs have been licensed to treat human influenza, but these antiviral drugs generally utilize an influenza-specific mechanism and therefore are not applicable to other viral infections. An important limitation of the currently licensed influenza drugs is that the virus evolves resistance easily [1], [2].

Favipiravir (or T-705) is a novel antiviral drug that is presently in clinical trials to treat human influenza [3]–[5]. It has a unique action among anti-influenza drugs in that it directly inhibits the viral RNA polymerase, but not cellular DNA or RNA polymerases [6], [7]. T-705 has shown *in vitro* and some *in vivo* inhibitory effects on a variety of pathogenic RNA viruses (examples include: Junin, Rift Valley Fever, Yellow Fever, West Nile, Western equine encephalitis, foot-and-mouth disease virus, and norovirus) [8]–[18]. These results are promising and exemplify the potential usefulness of T-705 as a broad-spectrum therapeutic.

Rift Valley Fever (RVF) is a globally-relevant, emerging disease in both livestock and humans in Africa and the Middle East, and outbreaks lead to millions of dollars of losses in human and economic terms in the affected areas [19], [20]. Due to the widespread range of the insect vectors, RVF virus (RVFV) has the potential to spread to Europe and the Americas [21]–[26], which would cause considerable economic damage and human morbidity and mortality. Although RVFV is a mosquito-borne virus, humans are largely exposed via contact or inhalation from contaminated animal carcasses. Aerosol exposure represents both a natural route of infection, and is also a route of concern for use of RVFV as a biological weapon. Therefore, evaluation of medical countermeasures against an aerosol exposure is an important component of pre-clinical studies.

There are currently no approved vaccines or therapeutics for preventing or treating RVF in either humans or ruminants. RVFV is listed as a Category A priority pathogen according to the National Institutes of Allergy and Infectious Diseases (NIAID) and is thus considered a potential bioterror threat due to the ease of transmission by inhalation [27]. It is regulated as an overlap Select Agent by the Centers for Disease Control (CDC) and U.S. Department of Agriculture (USDA) [28], [29] and requires biosafety level (BSL) -3 containment for laboratory and animal work with virulent isolates such as ZH501 [30].

Previous studies on the *in vitro* and *in vivo* efficacy of T-705 against RVFV have utilized either the MP-12 vaccine strain or the closely-related Punta Toro virus (both can be handled at BSL-2) as surrogates for wild-type BSL-3 strains of RVFV [9], [10]. In these previous studies, T-705 treatment showed *in vitro* activity against both viruses, as well as enhanced survival in mice and hamsters infected with Punta Toro virus.

Here, we evaluate the ability of T-705 to treat rats infected with the lethal and pathogenic ZH501 strain of RVFV. For this study, we utilize the Wistar-Furth rat model of inhalational Rift Valley Fever [31]. In this model, Wistar-Furth rats exposed to RVFV by aerosol develop a fulminant hepatic disease and succumb within 4–6 days post-infection. The median lethal dose (LD_50_) of aerosolized ZH501 RVFV in Wistar-Furth rats is 2 plaque-forming units (pfu), further emphasizing the sensitivity of this model. In this study, T-705 showed remarkable efficacy, with 92% of treated rats surviving lethal infection, even when T-705 was administered as late as 48 hours post-infection. This study provides important preclinical proof-of-concept data and demonstrates that T-705 is efficacious in a stringent rat model of lethal RVF. These findings support the broad-spectrum applicability of T-705 to treat pathogenic viral agents other than influenza.

## Materials and Methods

### Ethics statement

All animal work described here was carried out in strict accordance with the recommendations in the Guide for the Care and Use of Laboratory Animals of the National Institutes of Health and the Animal Welfare Act. The protocol was approved by the University of Pittsburgh Institutional Animal Care and Use Committee (Assurance Number A3187-01). The protocol was also approved by the Animal Care and Use Review Office of the US Army Medical Research and Materiel Command under protocol CB-2010-95.05. This work complies with Department of Defense (DoD) Instruction 3216.01 (Use of Animals in DoD Programs) and US Army Regulation 40-33 (The Care and Use of Laboratory Animals in DoD Programs).

### Biosafety information

All work with live RVFV was conducted at BSL-3 in the University of Pittsburgh Regional Biocontainment Laboratory (RBL). For respiratory protection, all personnel wore powered air purifying respirators (PAPRs) (either the 3M GVP-1 PAPR with L-series bumpcap, or 3M TR-3000 PAPR with 3M Versaflo M-Series headgear) or used a class III biological safety cabinet. All animals were housed in individually-ventilated micro-isolator caging (Allentown, Inc, Allentown, NJ). Vesphene II se (1∶128 dilution, Steris Corporation, Erie, PA) was used to disinfect all liquid wastes and surfaces associated with the agent. All solid wastes, used caging, and animal wastes, were steam-sterilized. Animal carcasses were digested via alkaline hydrolysis (Peerless Waste Solutions, Holland, MI). The University of Pittsburgh Regional Biocontainment Laboratory is a Registered Entity with the CDC/USDA for work with Rift Valley Fever.

### Virus propagation and culture

RVFV strain ZH501 was kindly provided by Barry Miller (CDC, Ft. Collins, CO) and Stuart Nichol (CDC, Atlanta). Prior to receipt, the virus was generated from reverse genetics plasmids [32] containing the wild-type ZH501 sequence, which was confirmed by sequencing. Virus was propagated on VeroE6 cells using standard methods. For virus quantitation, standard plaque assays were performed using an agarose overlay (1× minimum essential medium, 2% FBS, 1% penicillin/streptomycin, HEPES buffer, and 0.8% SeaKem agarose), incubated for 3 days at 37°C, and visualized using crystal violet. For titration of tissue samples, tissue pieces were homogenized in 2× volume of DMEM+10% FBS using an Omni tissue homogenizer (Omni International), followed by a standard plaque assay on the homogenate.

### Animal studies

Female Wistar-Furth rats (WF/NHsd; 8 to 10 weeks old) were obtained from Harlan Laboratories. All animals were provided rodent food (IsoPro Rodent 3000) and water ad libitum. All rats had implantable, programmable temperature transponders (IPTT-300; Bio Medic Data Systems, Seaford, DE) inserted subcutaneously between the shoulder blades for identification and temperature monitoring. Rats were exposed to RVFV as described below. Body temperature and weight were recorded daily starting 4 days prior to infection, and each animal was monitored at least twice daily for the development of clinical signs, including hunched posture, ruffled fur, decreased activity or response to stimuli, or neurological signs (head tilt and circling or rolling in cage). Rats meeting endpoint criteria as defined in the IACUC protocol we euthanized. Endpoint criteria vary depending upon the strain of rat infected with RVFV. For Wistar-Furth rats, which are very sensitive to RVFV, endpoint criteria include two or more of the following: body temperature of >39°C or <36°C, porphyrin staining around the eyes/nose/mouth, unresponsive to stimuli, or >5% weight loss for more than 2 consecutive days. Any rats that survives beyond 6 days post-infection and that also displays neurological signs (head tilt, tremors, circling or rolling in cage) along with weight loss (>5%) or temperature variations (>39°C or <36°C) also qualify for euthanasia. Blood was drawn by cardiac puncture immediately prior to euthanasia. During necropsy, a portion of each tissue was frozen for determination of viral load by plaque assay, and a second piece of each tissue was fixed in 10% neutral buffered formalin for 2 weeks prior to removal from the BSL-3 facility. Fixed tissues were processed for histology as described [33]. The pathologist was blinded when scoring samples for the degree of pathological changes.

### Aerosol exposures

Inside a Class III biosafety cabinet, rats were exposed for 10 minutes in a whole-body aerosol chamber to small-particle aerosols created by a 3-jet Collison nebulizer (BGI, Inc., Waltham, MA) controlled by the AeroMP aerosol exposure control system (Biaera Technologies, Hagerstown, MD). Air sampling and dose calculation were done as previously described [31].

### T-705 drug treatment

Favipiravir (T-705) was supplied by Toyama Chemical Company (Tokyo, Japan). Suspensions of T-705 were made using a mortar and pestle followed by dilution to the desired concentration in 0.4% carboxymethylcellulose (CMC) and 10% sucrose. Untreated control rats were given 0.4% CMC and 10% sucrose as a vehicle control. T-705 was administered orally to rats using a 1 ml syringe in a volume of 200 ul. While wearing bite-resistant gloves, rats were picked up and restrained by scruffing. The syringe was then inserted into the mouth towards the cheek, and the drug suspension was slowly ejected. All rats complied with drug administration.

### Indirect IgG ELISA

96-well plates were coated with RVFV MP-12-infected inactivated lysate (diluted 1∶20 in DPBS) and incubated overnight at 4°C. Plates were washed 3 times, followed by the addition of control or test sera (diluted 1∶100–1∶6400, four-fold, in blocking buffer) in duplicate. After incubation at 37°C for 1 hour, the plates were washed 3 times, followed by the addition of horseradish peroxidase-conjugated goat anti-rat IgG (H+L, KPL, Inc., Gaithersburg, MD) diluted 1∶2000 in blocking buffer. The secondary antibody was incubated for 1 hour at 37°C, washed 3 times, and 2,2′-azinodiethylbenzothiazoline sulfonic acid (ABTS) substrate (KPL, Inc.) was added to each well. Plates were incubated in the dark at 37°C for 30 minutes. After that time, 100 µL of ABTS stop solution was added to the wells and the optical density (OD) was determined at 405 nm. The SumOD was determined by subtracting the blank value from all wells, then summing the average ODs from each dilution (1∶100, 1∶400, 1∶1600, 1∶6400) for each sample.

### Statistical analyses

Statistics were performed using GraphPad Prism Software. Nonparametric logrank tests (Mantel-Cox) was used to compare survival distribution between T-705-treated groups and untreated control animals. Unpaired t-tests were used to compare the mean time to death (MTD) of treated and untreated groups of rats. Significance of ELISA results was determined by unpaired t-tests comparing each treated group with the untreated control group. Asterisks indicate significance as follows: **** = p<0.0001; *** = 0.0001<p<0.001; ** = 0.001<p<0.01; * = 0.01<p<0.05.

## Results

Wistar-Furth rats have historically been the most commonly used rat strain for studying RVF virus infections due to their rapid development of fulminant hepatitis after exposure to small doses of the pathogenic ZH501 strain [31], [32], [34]–[36]. The LD_50_ of RVFV ZH501 in Wistar-Furth rats is approximately 2 pfu for both subcutaneous and aerosol exposure [31], [37]. Death of Wistar-Furth rats occurs within 4–6 days after aerosol exposure. Moribund Wistar-Furth rats have high levels of infectious virus throughout a wide range of peripheral tissues (liver, lung, spleen, brain, heart, kidney), with the highest levels of virus replication occurring in the spleen and liver (10^8^ to 10^9^ pfu/g) [31]. Wistar-Furth rats typically do not display clinical signs until the last 24 hours before death, when a short febrile response, minimal weight loss, and porphyrin staining around the eyes/nose/mouth are hallmarks of end-stage disease.

Because they develop a precipitously lethal disease at a very low dose, Wistar-Furth rats represent a very stringent model in which to test potential vaccine and therapeutic candidates. They further serve as a relevant model of the acute hepatic/hemorrhagic disease seen in a subset of humans infected with RVFV [31], [38]–[40]. This study evaluated the effectiveness of T-705 to prevent illness and death in Wistar-Furth rats infected with the virulent ZH501 strain of RVF virus by inhalational exposure. Efficacy was evaluated in two experiments: a dose-determination study and a delayed-treatment study.

### T-705 dose-determination study

**Figure 1:**
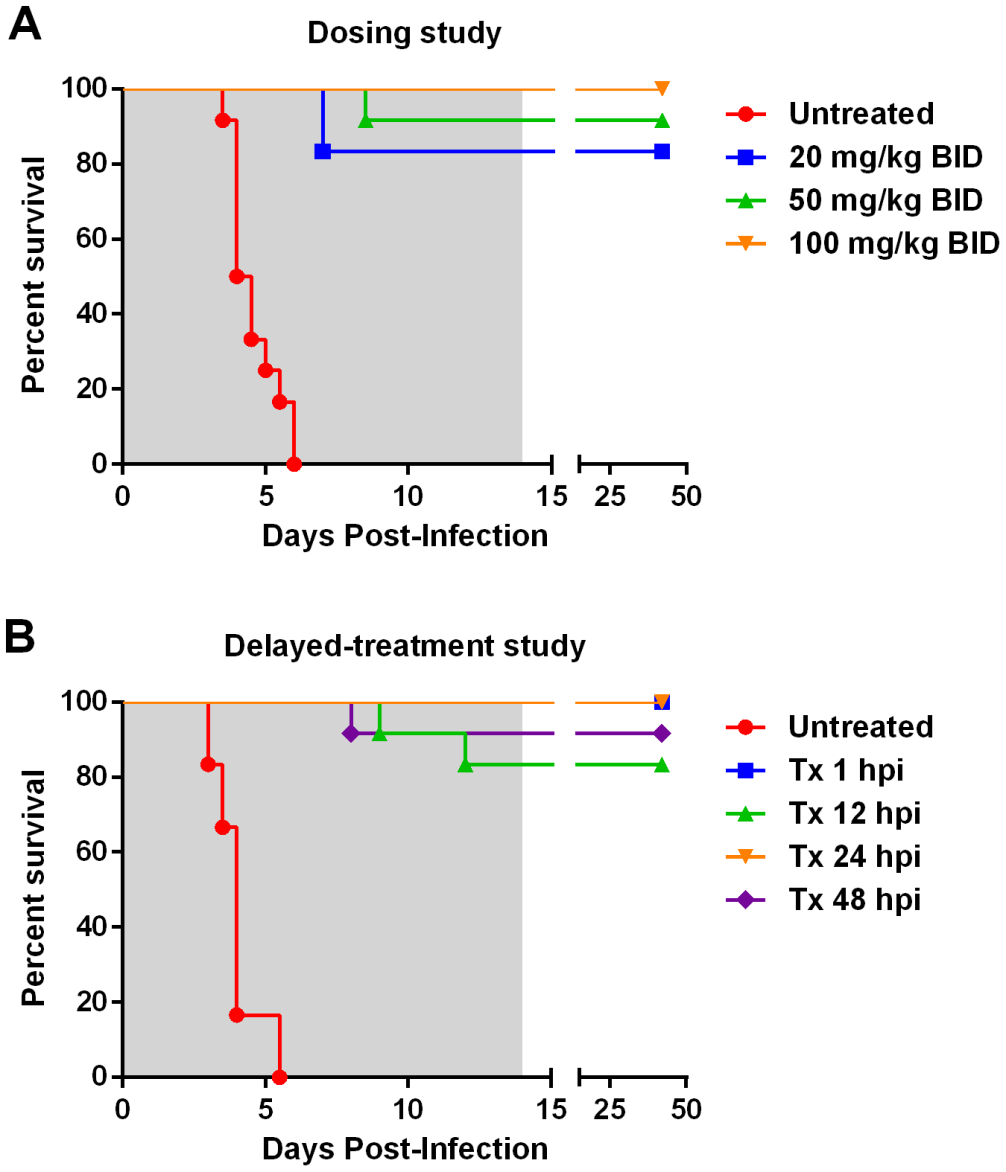
Survival of ZH501 RVFV-infected Wistar-Furth rats after treatment with T-705. (A) Results from T-705 dose-determination study. Groups of 12 rats were infected with RVFV by aerosol exposure, with an average presented dose of 50 pfu/rat. Within 1 hour after infection, twice-daily (BID) T-705 administration was given at the indicated doses. After 14 days (shaded area), drug treatment was removed, and the rats were monitored for another 28 days, as indicated by the split x-axis. (B) Results from T-705 delayed-treatment study. Groups of 6–12 rats were infected by aerosol exposure, with an average presented dose of 20 pfu/rat. All treated rats received 100 mg/kg of T-705 starting at the indicated times post-infection and continuing for 14 days (shaded area), followed by an additional 28 days of monitoring for survival (split x-axis). For ease of visual identification, the T-705 treatment period is indicated by gray shading from days 0–14 post-infection. For the delayed treatment groups, the treatment periods are shifted by 12, 24, or 48 hours, which is not indicated on the graph. In both experiments, all groups of T-705 treated rats survived significantly longer when compared to the untreated control rats (Log-Rank test p<0.0001).

The dose range of T-705 used in human clinical trials for treatment of influenza infection is between 50–100 mg/kg given orally twice or three times a day. Based on this, doses of 20, 50, and 100 mg/kg were administered to Wistar-Furth rats twice daily (BID) by the oral route following infection with RVFV. In the initial study, all rats received an average of 50 pfu of the virulent recombinant RVFV strain ZH501 during the aerosol exposure, which is approximately 25 LD_50_ [31]. At this challenge dose, all of the untreated control rats succumbed to infection within 4–6 days as expected (Fig. 1A). For the rats treated with T-705, the first dose was administered within 1 hour post-infection (hpi), and then treatment was continued twice daily for 14 days. After the end of treatment, the rats were monitored for survival for another 28 days.

Regardless of dose, T-705 treatment significantly prolonged survival of Wistar-Furth rats after RVFV challenge (Fig. 1A; p<0.0001 by Log-Rank test for each treated group compared to untreated controls). Two rats (out of 12) succumbed in the 20 mg/kg group, one rat (out of 12) succumbed in the 50 mg/kg group, and all rats given 100 mg/kg of T-705 survived. Of the three T-705 treated rats that succumbed, the mean time to death (MTD) was significantly longer than the untreated controls (7.5+/−0.5 days compared to 4.6+/−0.3 days, respectively; p = 0.0001 by unpaired t-test). Survival times were not significantly different between the groups that received T-705 as determined by Log-Rank test.

### T-705 delayed-treatment study

While T-705 displayed remarkable efficacy when given immediately after infection, an important question was whether T-705 would be useful as a post-exposure intervention when treatment was delayed. A second experiment was performed whereby initiation of T-705 treatment was delayed by 12, 24, or 48 hpi (Fig. 1B). All rats received a dose of 20 pfu of RVFV by aerosol exposure (approximately 10 LD_50_). All of the T-705-treated rats received 100 mg/kg BID starting at the indicated time points and continuing for 14 days. As expected, the RVFV-infected, untreated rats succumbed to infection with MTD of 4.0+/−0.3 days. Treatment with T-705 was remarkably effective even when drug administration was delayed until 48 hpi (Fig. 1B). All rats in the 1 hpi and 24 hpi groups survived. One rat (out of 12) in the 48 hpi group and two rats (out of 12) in the 12 hpi group succumbed to infection with MTD of 9.7+/−1.2 days. There was no significant difference in survival times between any of the T-705 treated groups, regardless of the time of initiation of treatment, but all treated groups survived significantly longer than the infected, untreated control rats (p<0.0001 by Log-Rank test for comparison between each treated group and untreated controls). Delaying T-705 treatment beyond 48 hpi was not evaluated.

### Clinical and virological assessment of rats treated with T-705

**Figure 2:**
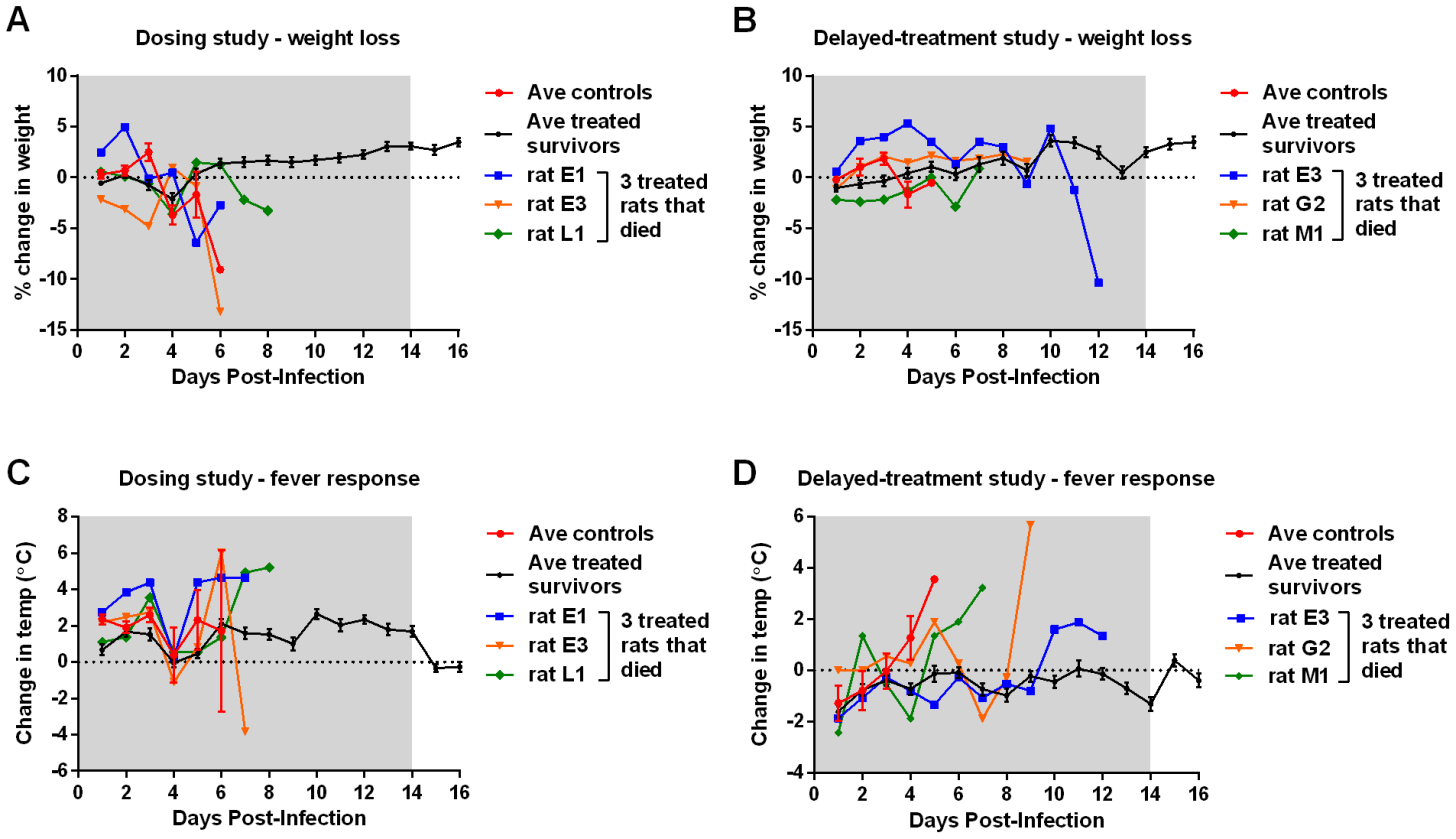
Weight loss and fever responses in Wistar-Furth rats treated with T-705. Results from the dose-determination study (A, C) and the delayed-treatment study (B, D) are shown. For each graph, the shaded area represents the 14 days of T-705 treatment. The black lines represent the mean +/− SEM of the infected, treated surviving rats (n = 33 in A and C; n = 39 in B and D). The red line represents the mean +/− SEM of the infected, untreated control rats (n = 12 in A and C; n = 6 in B and D). Three rats from each experiment were infected, treated, and succumbed to infection. Their weight loss and temperature data are plotted separately on each panel (blue, orange, and green lines). The x-axis is plotted through day 16 only in order to make the earlier time points more clearly visible.

Throughout the course of these studies, a total of 72 total rats were infected with RVFV and treated with T-705 at various doses and times post-infection, and only 6 succumbed to disease. The remaining 66 rats (92%) survived this highly lethal infection with no significant weight loss or fever (Fig. 2).

**Figure 3:**
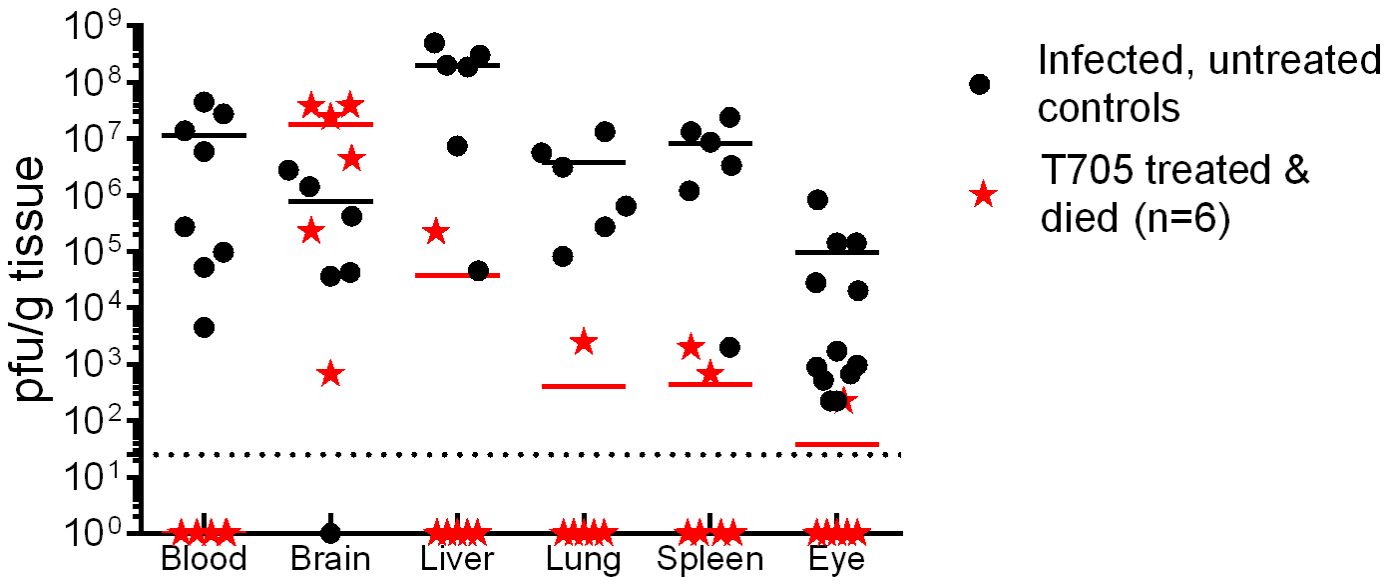
Tissue viral loads during T-705 efficacy studies in Wistar-Furth rats. Plaque assays were used to measure levels of infectious virus within the indicated tissues. Data from both the dose-determination study and the delayed-treatment study are combined in this figure. The black circles represent the tissue viral load from rats that were infected with RVFV but did not receive T-705 treatment (n = 6–12 per tissue; horizontal line is the mean). For the T-705-treated rats, 92% survived (n = 66), and tissues taken at necropsy from the surviving rats did not have detectable tissue viral loads by plaque assay (data not shown). A total of 6 T-705 treated rats from both studies died. The tissue viral loads from these 6 rats are shown as individual data points (red stars; red horizontal line is the mean).

**Figure 4:**
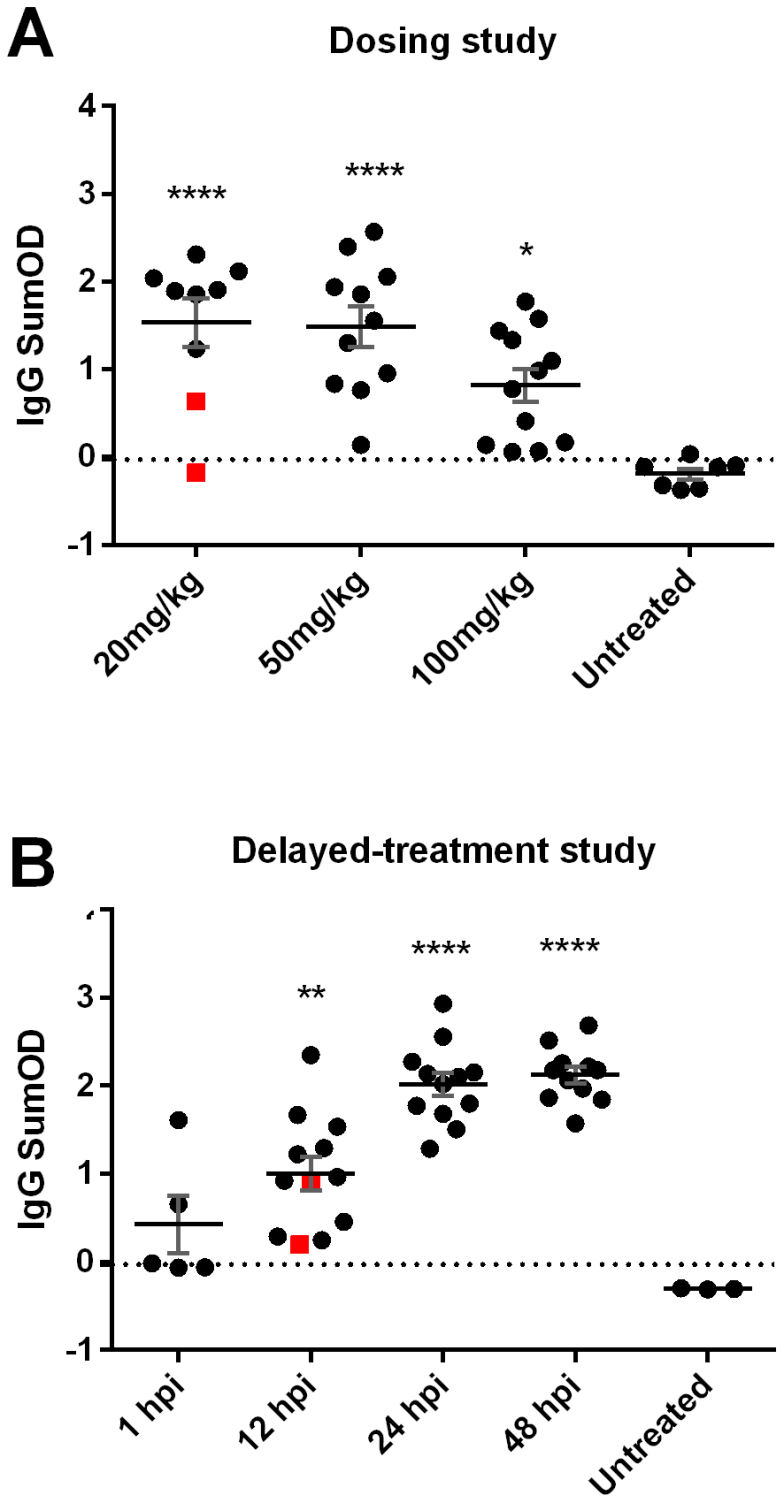
IgG antibody responses during T-705 efficacy studies in Wistar-Furth rats. Total IgG levels were measured using an indirect antigen-capture ELISA, and the data are expressed as the SumOD of each sample. The cutoff value is indicated by the horizontal dotted line, which represents 3 standard-deviations above the control wells. (A) Dose-determination study, and (B) Delayed-treatment study. For the untreated groups on each panel, serum was taken at necropsy when the rats were moribund (between 4–6 days post-infection). For the surviving rats within the T-705 treated groups (black circles), serum samples were taken at necropsy at the end of the study (day 42). Serum was available for 4 of the 6 T-705 treated rats that died (indicated by the red squares on each graph). Unpaired t-tests were used to compare each T-705 treated group with the untreated control group. Level of significance is indicated by the number of asterisks.

In both studies, the untreated RVFV-infected control rats lost weight and developed fever within the last 24 hours before death (Fig. 2, red lines). As expected based on our previous work [31], these untreated rats had high viral loads in peripheral tissues, including liver, spleen, and eye (Fig. 3, black circles). Virus-specific antibody responses as measured by a total IgG ELISA showed that the infected, untreated rats did not develop a detectable antibody response prior to succumbing to the infection (Fig. 4).

The 66 surviving rats from all T-705-treated groups (regardless of dose or time of treatment initiation) remained clinically healthy, with minimal weight loss or fever, even after T-705 treatment was removed (Fig. 2, black lines and data not shown). Surviving rats had no detectable infectious virus (as measured by plaque assay) in any tissues tested at necropsy (data not shown). Evidence of the curtailed infection was confirmed by an indirect IgG ELISA, with nearly all surviving rats displaying seroconversion at the time of necropsy (Fig. 4A and B, black circles). Interestingly, in both studies, several of the surviving rats treated with the highest dose of T-705 (100 mg/kg) immediately after exposure (1 hpi) had IgG antibody levels barely above the cutoff value, suggesting that T-705 abated virus replication in these animals, thereby preventing development of strong adaptive immune responses.

Within the dose-determination study (Fig. 4A), all 3 groups of T-705 treated rats had significantly higher levels of total IgG compared to the untreated control group (Fig. 4A, level of t-test significance indicted by asterisks). There was an inverse correlation between the average serum IgG titer and the dose of T-705 administered immediately after exposure (Fig. 4A). Rats given the lowest dose of T-705 presented with the highest serum IgG titers, whereas rats that received the highest dose of T-705 presented with the lowest IgG levels. This suggests that as the drug dose increased, less viral replication occurred and the antibody response was diminished.

For the delayed-treatment study, all 3 delayed groups had significantly higher IgG levels compared to the untreated controls, as indicated by the asterisks in Fig. 4B. There was also a concomitant inverse correlation between serum IgG titers and time of initiation of T-705 treatment (Fig. 4B). IgG titers were highest when the drug was administered at 48 hpi. IgG titers decreased as the treatment delay decreased, with several of the rats treated immediately after exposure remaining at the cutoff value (Fig. 4B).

The 6 treated rats that succumbed to infection over both studies had a significantly longer MTD than their infected, untreated counterparts (8.6+/−0.76 and 4.4+/−0.2 days, respectively when combining data from both experiments; p<0.0001 by t-test). Most of these 6 rats also displayed weight loss and all displayed body temperature alterations that were not seen in the 66 treated survivors (Fig. 2, individual data points). The delayed death in these 6 rats was clinically associated with neurological signs such as tremor, head-tilt, and circling in their cage. Typically, Wistar-Furth rats succumb to RVFV infection prior to the development of neurological signs; however there is evidence that a small proportion of infected Wistar-Furth rats that survive beyond day 7 die of neurological disease [41], which is reminiscent of what is seen in ACI and Lewis rats infected with RVFV [31]. Unlike the infected untreated control rats, virus was found primarily within the brain of these 6 treated rats that died with neurological signs, with much lower levels in peripheral tissues and none detectable in the blood at necropsy (Fig. 3, red stars). Blood samples were available from 4 of these 6 rats, and all 4 rats had serum IgG levels near the cutoff value (Fig. 4, red squares).

From a histological standpoint, infected, untreated control rats displayed pathological changes primarily in the spleen and liver, whereas the T-705 treated surviving rats had milder changes in these 2 tissues (Table 1). The 6 T-705-treated rats that died had very little pathology in the liver and spleen, but significant changes in the brain in the form of meningitis and lymphocytic vasculitis, which is concordant with the viral loads in the tissue (Fig. 3) and our previous data from rats that died with neurological disease [31].

**Table 1:** Pathological changes in T-705-treated Wistar-Furth rats.

Group	Liver pathology (apoptosis)	Spleen pathology (apoptosis in red pulp)	Meningitis	Lymphocytic Vasculitis in the brain
Infected, untreated controls	2.5+/−0.3#	1.8+/−0.4	0.75+/−0.5	0.0+/−0.0
T-705 treated survivors	1.0+/−0.2	1.0+/−0.3	0.3+/−0.2	0.0+/−0.0
T-705 treated died	0.0+/−0.0	0.5+/−0.2	2.4+/−0.2	1.6+/−0.5

# = average score of the degree of severity of pathological lesions, where 0 = no pathology and 3 = severe pathology; +/− SEM.

## Discussion

Broad-spectrum antivirals represent a gold-standard in the development of effective countermeasures for treating a wide range of viral diseases. The most well-known broad-spectrum drug, Ribavirin, is currently FDA-approved to treat hepatitis C in combination with interferon. Aerosolized ribavirin may be an effective treatment for respiratory syncytial virus infections and as an off-label use for treatment of hemorrhagic fever viruses such as Lassa. Ribavirin has shown *in vitro* and *in vivo* activity against a number of viruses, including RVFV, but success at treating infected patients is mixed [42], [43]. The clinical utility for the treatment of RVF with Ribavirin has not been thoroughly tested and is only recommended under compassionate use guidelines [42], [44]–[50]. Recent studies directly comparing Favipiravir activity with Ribavirin suggest that Favipiravir is less toxic while having equal or greater efficacy [8].

Wistar-Furth rats are very sensitive to RVFV when administered subcutaneously or by aerosol [31], [35]–[37], [41]. Here, we utilized an aerosol infection model which results in uniform lethality from a fulminant hepatic disease within 4–6 days post infection [31]. Clinical manifestations of this aerosol model include fever, weight loss, porphyrin staining around the eyes/nose/mouth, ruffled fur, and hunched posture. When Wistar-Furth rats succumb to RVFV, virus is widespread in peripheral tissues, including liver, spleen, kidney, lung, and heart. In the current study, rats treated with T-705 survived infection with minimal weight loss or fever, no detectable virus in the tissues at necropsy, minimal tissue pathology, and measurable virus-specific antibody responses. T-705 was able to abate virus replication enough that the vast majority (92%) of rats survived infection without obvious morbidity or mortality.

The small number of treated rats that died (6/72) developed delayed-onset neurological disease as indicated by clinical signs, pathology, and viral load in the brain tissue. Wistar-Furth rats typically develop early-onset hepatic disease, although it has been reported previously that vaccinated Wistar-Furth rats that succumbed to aerosolized RVFV died later than expected with encephalitic disease [41]. Similarly, mice that survive the early hepatic form of disease after RVFV infection succumb to delayed-onset neurologic disease [51]–[53]. The mechanism of neuroinvasion in inhalationally-exposed rats is under investigation, but work with mice has shown that the olfactory bulb may play a critical role [51], [53], similar to what has been shown for other arboviruses [54], [55].

An aerosol infection model represents a stringent framework for evaluation of novel therapeutics. Studies with mice have shown that Ribavirin provides partial prophylactic protection from subcutaneous infection with RVFV but no protection from aerosol challenge [53]. This study highlights the need for antivirals that will protect from aerosol exposure, which is the most likely method for dispersal of a biological weapon [56]. Here we show that Favipiravir (T-705) is remarkably effective at preventing disease and death in Wistar-Furth rats exposed to aerosols of the virulent ZH501 strain of RVFV.

There are a number of challenges to developing novel antiviral therapeutics for emerging diseases and biodefense agents such as Rift Valley Fever. In 2002, the FDA published the Animal Rule which was designed to help respond to potential biological and chemical threats for which efficacy trials in humans were neither ethical nor logistically possible [57]. The Animal Rule states that adequate and well-controlled animal trials can substitute for human clinical trials in certain specific situations outlined in the Animal Rule. To date, no antiviral drugs or vaccines have been approved for human use solely based on the Animal Rule. As a result of ongoing phase III efficacy trials for use of T-705 to treat influenza, this drug is well-characterized for safety in humans. Therefore it is possible that T-705 may be analogous to three previous cases where drugs approved for separate indications become approved for new indications through the use of the Animal Rule [58]–[61]. Of these three drugs, one was a new clinical indication for an already approved drug (pyridostigmine bromide to treat soman nerve agent), the second for a drug approved in Europe (hydroxocobalamin for cyanide poisoning), and the most recent was for the use of a previously approved antibiotic, levofloxacin, to treat pneumonic plaque [62]. The limitations of the Animal Rule are well-described, and it is clear that it is not a path to quick approval [58]. However, based on the 3 examples cited above, obtaining a label-extension or new indication for an already licensed drug is probably the most realistic route of approval for drugs to treat emerging or highly pathogenic viruses.

The effectiveness of T-705 against RVFV and other viruses *in vitro* and *in vivo* makes this novel drug a promising broad-spectrum candidate. This study provides important preclinical proof-of-concept data demonstrating that T-705 is efficacious in a stringent rat model of lethal inhalational RVF even when treatment was delayed 48 hours post-exposure. Further evaluation of the efficacy of T-705 against the pathogenic strain of RVFV in a non-human primate model is warranted. These findings are an encouraging step forward and support the applicability of T-705 to treat pathogenic viral agents other than influenza. Additional animal studies with pathogenic strains of other medically important viruses are warranted.
